# Supplementary material for: Simultaneous Quantification and Speciation of Trace Metals in Paired Serum and CSF Samples by Size Exclusion Chromatography–Inductively Coupled Plasma–Dynamic Reaction Cell–Mass Spectrometry (SEC-DRC-ICP-MS)
Source: Int J Mol Sci. 2021 Aug 18;22(16):8892. doi: 10.3390/ijms22168892 (PMC8396360; doi:10.3390/ijms22168892)
Supplement: Supplementary file 1 [file ijms-22-08892-s001.zip › ijms-1319634-supplementary.pdf]

Supplementary figures to

Simultaneous speciation of trace metals in paired serum and CSF samples by size exclusion chromatography–inductively coupled plasma–dynamic reaction cell–mass spectrometry (SEC-DRC-ICP-MS)

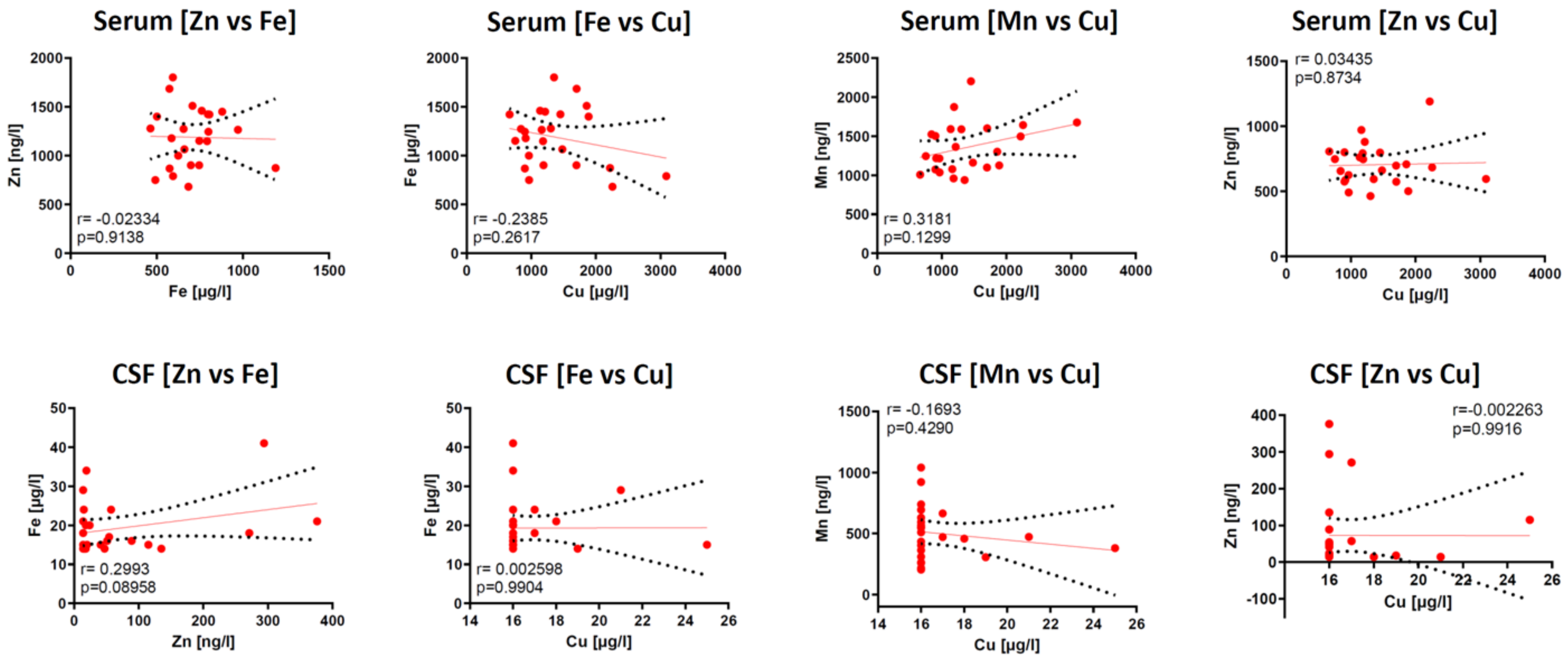

Supplemental Figures S1:

Figure title: Pearson's correlation analysis: element vs. element interactions in serum and CSF

Pearson's correlation analysis are shown for element vs. element interactions in serum and CSF. There was no correlation found for any of the here shown element pairs, neither in serum nor CSF.

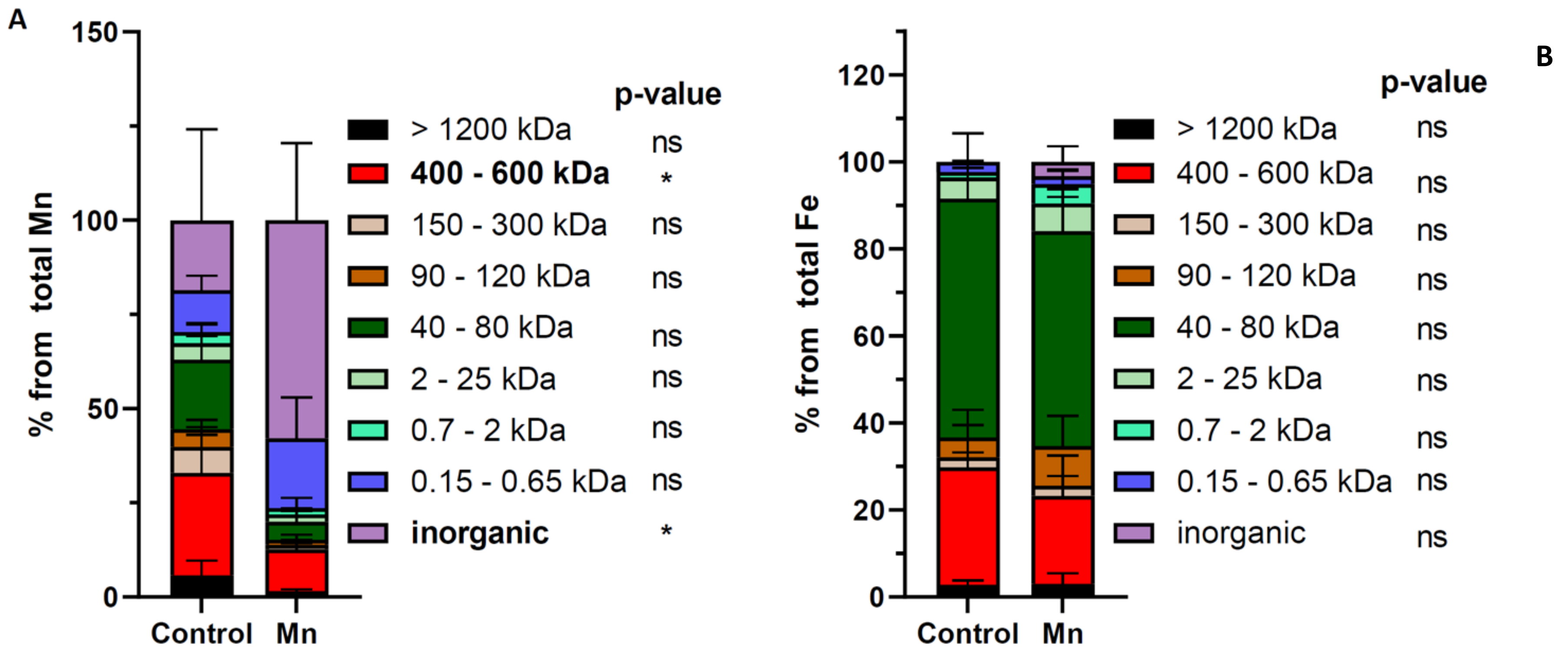

Supplemental Figures S2:

Figure title: Comparison of element concentrations in size fractions

Comparison of element concentrations in size fractions from brain extracts of control rats or Mn-exposed rats after a single i.v. $MnCl_2$ injection (100 µL of 1.5 mg Mn/kg body weight).

A) Changes of the manganese concentrations: After a single i.v. $MnCl_2$ injection the Mn-concentration in the 400-600 kDa fraction (supposedly α-2-macroglobuline) is significantly decreased, whereas the inorganic fraction is drastically increased.

B) No changes of the iron concentrations: After a single i.v. $MnCl_2$ injection in none of the size fractions a significant change in Fe-concentrations was observed.
